# DARS: a phase III randomised multicentre study of dysphagia- optimised intensity- modulated radiotherapy (Do-IMRT) versus standard intensity- modulated radiotherapy (S-IMRT) in head and neck cancer

**DOI:** 10.1186/s12885-016-2813-0

**Published:** 2016-10-06

**Authors:** Imran Petkar, Keith Rooney, Justin W. G. Roe, Joanne M. Patterson, David Bernstein, Justine M. Tyler, Marie A. Emson, James P. Morden, Kathrin Mertens, Elizabeth Miles, Matthew Beasley, Tom Roques, Shreerang A. Bhide, Kate L. Newbold, Kevin J. Harrington, Emma Hall, Christopher M. Nutting

**Affiliations:** 1 The Royal Marsden NHS Foundation Trust, Fulham Road, London, SW3 6JJ UK; 2 The Institute of Cancer Research (ICR), 123 Old Brompton Road, London, SW7 3RP UK; 3 Northern Ireland Cancer Centre, Belfast Health and Social Care Trust, Belfast City Hospital, Lisburn Road, Belfast, BT9 7AB UK; 4 Speech and Language Therapy Department, Sunderland City Hospitals NHS Foundation Trust, Kayll Road, Sunderland, SR4 7TP UK; 5 Institute of Health and Society, University of Newcastle, Newcastle upon Tyne, NE1 7RU UK; 6 Mount Vernon Hospital, Rickmansworth Road, Northwood, HA6 2RN UK; 7 University Hospitals Bristol, Horfield Road, Bristol, BS2 8ED UK; 8 Norfolk and Norwich University Hospital NHS Trust, Colney Lane, Norwich, NR4 7UY UK

**Keywords:** Dysphagia, Pharyngeal cancer, Dysphagia-optimised intensity-modulated radiotherapy, Pharyngeal constrictor muscle

## Abstract

**Background:**

Persistent dysphagia following primary chemoradiation (CRT) for head and neck cancers can have a devastating impact on patients’ quality of life. Single arm studies have shown that the dosimetric sparing of critical swallowing structures such as the pharyngeal constrictor muscle and supraglottic larynx can translate to better functional outcomes. However, there are no current randomised studies to confirm the benefits of such swallow sparing strategies. The aim of Dysphagia/Aspiration at risk structures (DARS) trial is to determine whether reducing the dose to the pharyngeal constrictors with dysphagia-optimised intensity- modulated radiotherapy (Do-IMRT) will lead to an improvement in long- term swallowing function without having any detrimental impact on disease-specific survival outcomes.

**Methods/design:**

The DARS trial (CRUK/14/014) is a phase III multicentre randomised controlled trial (RCT) for patients undergoing primary (chemo) radiotherapy for T1-4, N0-3, M0 pharyngeal cancers. Patients will be randomised (1:1 ratio) to either standard IMRT (S-IMRT) or Do-IMRT. Radiotherapy doses will be the same in both groups; however in patients allocated to Do-IMRT, irradiation of the pharyngeal musculature will be reduced by delivering IMRT identifying the pharyngeal muscles as organs at risk. The primary endpoint of the trial is the difference in the mean MD Anderson Dysphagia Inventory (MDADI) composite score, a patient-reported outcome, measured at 12 months post radiotherapy. Secondary endpoints include prospective and longitudinal evaluation of swallow outcomes incorporating a range of subjective and objective assessments, quality of life measures, loco-regional control and overall survival. Patients and speech and language therapists (SLTs) will both be blinded to treatment allocation arm to minimise outcome-reporting bias.

**Discussion:**

DARS is the first RCT investigating the effect of swallow sparing strategies on improving long-term swallowing outcomes in pharyngeal cancers. An integral part of the study is the multidimensional approach to swallowing assessment, providing robust data for the standardisation of future swallow outcome measures. A translational sub- study, which may lead to the development of future predictive and prognostic biomarkers, is also planned.

**Trial registration:**

This study is registered with the International Standard Randomised Controlled Trial register, ISRCTN25458988 (04/01/2016)

## Background

Cancer of the pharynx affects around 3000 patients in the UK annually [1], with a majority of cases caused by infection with human papillomavirus (HPV) [2]. For most newly diagnosed patients organ-preserving CRT or radiation alone is the treatment of choice. A significant proportion of survivors, however, subsequently suffer from long-term treatment- related toxicities such as xerostomia and dysphagia. Improving such functional outcomes is pivotal in an era where younger and healthier patients are increasingly cured of their HPV- driven tumours with CRT [3], only to be exposed to decades of debilitating radiation- induced morbidity resulting in an adverse impact on health- related quality of life (HR-QoL). There has been a renewed focus recently to address this issue, with the required impetus to achieve this goal facilitated by the widespread availability of advanced radiation delivery techniques.

Dysphagia following CRT represents a substantial problem, with nearly 50 % of patients identifying it as a distressing symptom following radiation treatment [4]. Radiation dose to critical structures involved in the swallowing mechanism and post- radiation pharyngo-oesophageal strictures contribute significantly to poor long-term function. A major clinical consequence of swallowing dysfunction is aspiration and related pneumonia [5–8]. This is typically under-reported in most head and neck cancer (HNC) trials, where assessments are undertaken only at the onset of clinical symptoms only, thereby failing to detect the silent aspirators [9]. Dietary modifications, nutritional deficiencies, and prolonged feeding tube dependence [10, 11] are usually a consequence of persistent dysphagia, resulting in poor social interactions along with lifestyle alterations for both patients and their carers/family members [12]. Finally, late radiation- associated dysphagia is a distinct entity characterised by a delayed onset of swallowing dysfunction in combination usually with lower cranial neuropathy, which invariably leads to aspiration pneumonia in a majority with subsequent lifelong dependence on a feeding tube [13].

It is evident that dysphagia following CRT has a negative impact on a patient’s physical, social and emotional state. Yet, consistent, prospective evaluation of all three states of swallowing outcomes is conspicuous by its absence in most HNC studies reporting on post- treatment functional status [14]. Frequently used subjective tools, such as patient- reported outcomes and clinician- rated scores, provide invaluable information about HR-QoL and represent a quick, cost effective method of reporting swallowing outcomes. Toxicity reporting measures are, however, subject to significant inter-observer variability [15–17] and are also insensitive in quantifying functional abnormalities such as the risk of aspiration, which is detected using instrumental swallowing assessments such as videofluoroscopy (VF) or Fibreoptic Endoscopic Evaluation of Swallowing (FEES). Such variations in outcome reporting result in different normal tissue complication (NTCP) models predicted for dysphagia in the same patient population [18]. Lack of a comprehensive swallowing assessments necessitates caution in interpretation of the reported outcomes; particularly as the true burden of dysphagia- related morbidity might not have been accurately determined.

The introduction of intensity- modulated radiotherapy (IMRT) in HNC has improved HR-QoL by improving salivary function [19], and can reduce the delivered dose to critical swallowing structures [20]. In a pioneering study, a strong association was established between irradiation of the pharyngeal constrictor muscle (PCM), glottis and supraglottic larynx (SGL) and subsequent swallowing dysfunction [20]. To improve functional outcomes, it is imperative to safely spare these dysphagia/aspiration at risk structures (DARS). Numerous planning studies have confirmed a significant relationship between irradiation of various swallowing structures and persistent dysphagia [11, 21–29]; with the mean dose to the PCM a strong predictor of swallowing impairment in a systematic review [30]. Despite this, there is significant uncertainty regarding the clinically relevant structural and dosimetric predictors of long-term functional impairment. Differences in influential variables such as primary tumour location, tumour stage, use of concomitant chemotherapy, fractionation schedules, and in primary endpoints and target volume definition limit the conclusions that can be drawn. Furthermore, small sample sizes together with the retrospective nature of most studies and inconsistent swallow outcome recording affect the robustness of the reported results.

Promising results have emerged from prospective non-randomised, oropharyngeal cancer only studies. Feng et al. evaluated the efficacy of swallow- sparing chemo-IMRT in 73 patients with stage III/IV oropharyngeal cancers [31]. The IMRT technique involved sparing the PCM and SGL in the region of the rarely involved medial retropharyngeal lymph nodes (RPN), delivered by setting a dosimetric constraint of <50 Gy. Mean doses of 48 Gy and 42 Gy were achieved for the spared parts of PCM and SGL respectively; with corresponding mean doses to the entire organs of 58 Gy and 48 Gy. Crucially, this dosimetric sparing did not increase the risk of loco-regional recurrence, with no relapses observed within or near the spared structures. Long-term swallowing outcomes with this novel IMRT approach were only slightly worse compared to baseline, suggesting potential functional improvements. Subsequent dosimetric analysis revealed a significant association between worsened swallowing outcomes and mean doses to the entire PCM and its individual parts, particularly the superior constrictor, SGL and oesophagus [18]. Another smaller phase II study in oropharyngeal cancer has also demonstrated improved objective function by sparing the anterior oral cavity and the upper pharyngeal musculature [32].

Validated predictive models for RTOG ≥ grade 2 dysphagia at 6 months in a heterogeneous group of HNC patients treated with (chemo) radiation have also been developed by a consortium of Dutch radiation oncologists. Initial planning work found the superior PCM and SGL to be the strongest dosimetric predictors, with a subsequent *in-silico* study demonstrating likely improvements in the clinician- rated scores by safely minimising as much as possible the dose to the two swallowing organs at risk (OAR) without compromising target coverage. Finally, patients followed up prospectively showed clinically relevant functional improvements with this strategy [33–36]. As mentioned previously, the use of only a clinician-rated score to develop their model is a limitation of this study.

## Rationale for the DARS trial

Despite the available published literature regarding optimisation of radiotherapy techniques to improve long- term swallow function, the question of whether function- sparing IMRT techniques truly improve function and HR-QoL remains unanswered. In a world of evidence-based medicine, outcomes from single-arm studies confirming the existence of a strong association between dosimetric sparing of DARS and functional improvement are insufficient to alter current standards-of-care. Results from these studies strengthen the rationale to explore this important question within the context of a randomised controlled trial (RCT). The DARS trial (CRUK/14/014) was conceived primarily to answer this vital patient- centred question of whether the implementation of Do-IMRT in pharyngeal cancers will lead to a subjective improvement of swallow function.

In order to achieve satisfactory long-term swallowing function, it is necessary to acknowledge that a ‘one size fits all’ model to develop a swallow-sparing strategy for all H&N sub sites is unlikely to be successful. Although attempts should be directed to reducing the radiation dose to all swallowing OARs as much as clinically acceptable, sparing the swallowing structure in close proximity to the primary tumour should be a priority as it is a key determinant of subsequent poor functional outcome [21]. Therefore, establishing different dose constraints for the swallowing OARs, dependent on the location of the primary tumour, is essential. Evidence to date, confirms that a strong and clinically relevant correlation exists between PCM irradiation and the development of persistent dysphagia in pharyngeal cancers.

## Methods/design

### Study design

**Fig. 1:**
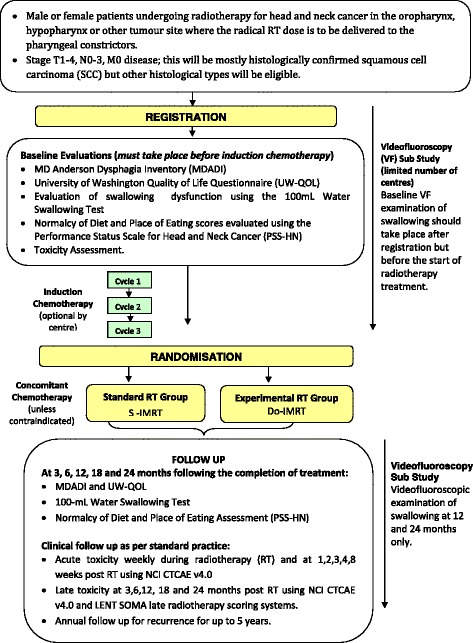
TRIAL SCHEMA

DARS is a parallel-group, multicentre phase III RCT, with blinded assessments of key outcome measures, in patients undergoing radical primary chemoradiation or radiation alone for pharyngeal tumours. Suitable patients will be randomised to either S-IMRT or Do-IMRT (Fig. 1). Radiotherapy dose and fractionation will be the same in both treatment groups; in the Do-IMRT arm, the dose to the PCM will be reduced by identifying it as an organ at risk, thereby optimising the treatment plan to meet specified dose constraints.

The trial opened to recruitment in June 2016 and is expected to recruit in approximately 20–25 UK centres.

#### Patient population

Patients should satisfy all the inclusion criteria and meet none of the exclusion criteria specified in Table 1 to be eligible for the trial. In brief, patients will have biopsy proven, pharyngeal cancers that will be treated with bilateral neck irradiation. The use of both prophylactic and reactive feeding tube insertions is acceptable, though patients would be encouraged to keep swallowing (even if very limited amounts) for the duration of treatment.

**Table 1:** Inclusion and exclusion criteria for patient recruitment in the DARS trial

ᅟ	ᅟ
Inclusion Criteria:	
• Aged 18 or above;	
• Any patient undergoing radiotherapy for HNC in the oropharynx or hypopharynx. Patients with tumour at other sites where radical radiotherapy dose is to be delivered to the pharyngeal constrictors may also be eligible;	
• Stage T1-4, N0-3, M0 disease; this will be mostly histologically confirmed squamous cell carcinoma but other histological types may be eligible;	
• Radiotherapy with concomitant chemotherapy (unless contraindicated) is the planned treatment;	
• Creatinine clearance (≥50 mL/min prior to starting chemotherapy); not applicable for patients receiving radiotherapy alone;	
• WHO performance status 0 or 1;	
• Available to attend long term follow- up;	
• Adequate cognitive ability to complete the MD Anderson Dysphagia Inventory (MDADI), University of Washington Quality of Life (UW-QoL) v.04 questionnaire and Performance Status Scale for Head & Neck Cancer (PSS-HN) assessments;	
• Written informed consent.	
Exclusion Criteria:	
• Documented evidence of pre-existing swallowing dysfunction (not related to HNC);	
• Previous radiotherapy to the head and neck region;	
• Posterior pharyngeal wall, post- cricoid and retropharyngeal lymph node involvement;	
• Lateralised tumours, requiring unilateral irradiation	
• Major head and neck surgery (excluding biopsies/tonsillectomy);	
• Current/previous tracheostomy placement;	
• Previous or concurrent illness, which in the investigator’s opinion would interfere with completion of therapy, trial assessments or follow-up;	
• Any invasive malignancy within previous 2 years (other than non-melanomatous skin carcinoma or cervical carcinoma in situ).	

#### Study objectives and endpoints

The primary objective of the DARS trial is to determine whether reducing the radiation dose to DARS using Do-IMRT, improves swallowing function compared to S-IMRT in pharyngeal cancer patients treated with radical chemoradiation or radiation alone. The impact of the dosimetric sparing achieved with Do-IMRT on late swallowing function will be evaluated by a patient-reported outcome (PRO) using the MDADI. The difference in the mean MDADI composite score at 12 months after treatment completion between randomised treatment groups forms the primary endpoint of the trial.

Secondary objectives are to:

1. Investigate the longitudinal pattern of patient-reported swallowing function up to 2 years post- radiotherapy treatment using the MDADI;
2. Investigate the impact of using Do-IMRT on
  (i) normalcy of diet and public eating using the PSS-HN scale;
  (ii) swallowing performance using the 100 mL Water Swallow Test and VF examination (subset of centres only);
  (iii) acute and late toxicity
  (iv) duration of feeding tube use;
3. Assess patient-reported QoL and priority concerns using the UW-QoL questionnaire (v.04);
4. Compare cancer- related outcomes according to radiotherapy technique used, including resection rates, location and timing of loco-regional recurrence and overall survival.

#### Registration/randomisation

The trial has a two-stage entry process of registration and randomisation. Randomisation only occurs following target outlining, ensuring consistency across both the experimental and standard treatment volumes by avoiding any potential bias that could be introduced by the clinician during delineation. Additionally, patients and SLTs will be blinded to the treatment allocation to avoid bias during assessments. This is particularly relevant in a trial of this nature where a majority of endpoints rely on a combination of patient- reported outcomes and SLT- led evaluations.

Patients will be randomised between the 2 treatments on a 1:1 basis using the method of minimisation with a random element. Randomisation will be performed centrally by the Institute of Cancer Research Clinical Trials Statistics Unit (ICR-CTSU). Patients will be stratified prior to randomisation by centre, use of induction and concomitant chemotherapy, tumour site (incorporating HPV status for oropharyngeal tumours) and American Joint Committee on Cancer (AJCC) tumour stage.

#### Chemotherapy

Induction chemotherapy is optional and will follow the centres’ standard policy; a maximum of 3 cycles of platinum-based chemotherapy can be administered prior to radiotherapy. The principal investigator of each centre will be expected to define their use of induction chemotherapy by TNM stage and tumour site prior to the trial opening.

Concomitant chemotherapy is recommended for all patients, unless there is a contraindication, in which case radiotherapy alone will be permitted. The standard regimen will be cisplatin 100 mg/m^2^ administered on day 1 and day 29 of the radiotherapy schedule; alternatively 50 mg/m^2^ on days 1 and 2 repeated again on days 29 and 30 will be acceptable. Carboplatin (AUC 5) can be substituted if patients have an existing co-morbidity or subsequently develop cisplatin- related toxicity.

#### Radiotherapy

Patients in both treatment groups will receive 65Gy in 30 fractions to the primary and nodal tumour (PTV_6500) and 54Gy in 30 fractions (PTV_5400) to the areas considered at risk of harbouring microscopic disease. Treatment will be delivered by a variety of IMRT techniques. Patients in the S-IMRT control group will receive the current standard- of- care radiation planning, whereas PCM irradiation will be reduced by introducing it as an OAR in the treatment planning objectives of patients allotted to the Do-IMRT arm.

Treatment verification will include the following as a minimum: orthogonal kilovoltage (KV) or megavoltage (MV) isocentre images, or cone beam CT images, taken on days 1–3 and then weekly. Any treatment gaps will be managed as per the Royal College of Radiologists guidelines for Category 1 patients, aiming to complete radiotherapy within 6 weeks.

#### Target volume delineation

DARS has adopted a volumetric approach to define the target volumes. Findings at the time of endoscopy along with pre-therapy imaging will be used to aid accurate delineation of the primary tumour. Two clinical target volumes (CTV) will be defined and edited to exclude natural barriers to disease spread. CTV_6500 will include the primary and nodal gross tumour volume (GTV) with a 1 cm isotropic margin while the prophylactic CTV_5400 will include the remainder of the involved subsite and nodal levels at risk of microscopic disease. Corresponding planning target volumes (PTVs) will be grown with 3–5 mm margins, according to the practice of individual centres. CT delineation of nodal levels will follow the recently updated outlining guidelines [37].

The superior and middle constrictors will be contoured as one structure (SMPCM) in the trial with the inferior PCM (IPCM) delineated as a separate structure. Outlining for the PCM is based on the published contouring guidelines defined by Christianen et al. in conjunction with the atlas produced for the Post-operative adjuvant treatment for HPV positive tumours (PATHOS; NCT02215265) trial [38, 39]. Other OARs will include the spinal cord, brainstem and the parotid glands.

#### Do-IMRT

The experimental Do-IMRT technique aims to spare the PCM lying outside the high dose CTV. For oropharyngeal primaries, mandatory mean dose constraints of <50 Gy to the volume of SMPCM lying outside CTV_6500 (PlanSMPCM) together with an optimal mean dose constraint of <20 Gy to the volume of IPCM lying outside CTV_6500 (PlanIPCM) have been defined. Likewise, for hypopharyngeal tumours, mandatory and optimal mean dose constraints of <50 Gy and <40 Gy have been set for PlanIPCM and PlanSMPCM respectively.

Crucially, it is important to note that although the PCM will overlap with the PTVs, there will be no sparing of the constrictor muscles that lie within the PTV_6500.

Planning objectives will be prioritised in the following order: critical organ constraints (spinal cord and brainstem); PTV_6500 coverage; constrictor constraints; PTV_5400 coverage; parotid gland constraints and other non-specified normal tissue.

#### Assessments

##### Toxicity and response assessments

NCI CTCAE v4.0 will be used to assess acute toxicity data that will be collected weekly during radiotherapy, and at week 1–4 and 8 after treatment completion. Late toxicity will be scored using both NCI CTCAE v4.0 and LENT SOMA scoring systems. Clinical assessments will be made at 6 weeks, and 6, 12, 18 and 24 months after completion of treatment as a minimum. Additional investigations will be requested if clinically indicated. Imaging response will be carried out at 3 months after radiotherapy and reported as per RECIST criteria v1.1. Patients found to have persistent cervical lymphadenopathy will proceed to neck dissection. Late toxicity and survival data will be collected at 3, 6, 12, 18 and 24 months post- treatment, after which routine follow up data will be collected annually for up to 5 years.

##### Swallowing assessments

A panel of subjective and objective swallowing outcome measures (Table 2) will assess swallow function at regular intervals.

**Table 2:** Functional measures and endpoints

Time point	Study	Domain	Endpoint
Baseline, 3, 6, 12, 18 and 24 months	MDADI	Swallowing related QoL	Composite (total), global, emotional, functional and physical subscale scores
Baseline, 3, 6, 12, 18 and 24 months	WST	Swallow Performance	Swallow capacity, Swallow volume
Baseline, 12 and 24 months	VF^a^	Airway protection	Penetration Aspiration Scale [52]
Baseline, 12 and 24 months	VF^a^	Physiology	MBSImp
Baseline, 12 and 24 months	VF^a^	Pharyngeal dysphagia grade	DIGEST grade [53]
Baseline, 3, 6, 12, 18 and 24 months	PSS-HN	Functional Performance Status	Normalcy of diet, eating in public, understandability of speech scores
Baseline, 3, 6, 12, 18 and 24 months	UW-Qol v.04	HR-QoL	Composite scores of physical and social-emotional functioning are derived from 12 domains. Patients can also highlight up to 3 priority concerns from the previous 7 days

Abbreviations: *WST* Water Swallowing Test, *DIGEST* Dynamic Imaging Grade of Swallowing Toxicity, *MBSImp* Modified Barium Swallow Impairment Profile

^a^Subset of centres only

#### Videofluoroscopy sub study

Instrumental swallowing assessment with VF will be performed in approximately 5–10 centres including up to 50 patients. The VF will be conducted by SLTs (with the required level of competency as set out by the Royal College of SLT guidelines) supported by a radiographer and/or radiologist. Central review and rating of images will be carried out by 2 clinical-academic SLTs (JWGR and JMP)

#### Translational sub study

The primary objectives of this planned sub- study are to obtain DNA and RNA from formalin-fixed, paraffin- embedded tumour sample for genomic analysis and to measure and quantify circulating tumour DNA (ctDNA) at various time points before and after treatment.

Secondary objectives include the determination of the sensitivity and specificity of ctDNA in predicting residual disease following treatment and recurrent disease during follow- up.

#### Statistical design

##### Sample size

In a previous cohort of patients treated with S-IMRT, mean MDADI composite score 12 months after treatment completion was 72 (SD = 13.8) [4]. A 10-point improvement in the MDADI composite score at 12 months is considered a clinically relevant outcome [40]. To have a 90 % power to detect this improvement (two- sided 5 % significance), 41 patients are required in each treatment group. Assuming a 20 % drop out due to disease recurrence, deaths and non-compliance with the 12-month questionnaire, the aim is to recruit 102 patients. MDADI compliance rates will be monitored regularly to ensure data on 82 evaluable patients are available.

Up to 50 of the recruited patients (25 in each arm) will additionally be included in the VF sub study. This will give 80 % power (one- sided significance 5 %) to detect an absolute difference of 33 % in the number of patients experiencing swallowing impairment according to the Penetration Aspiration Scale (based on 50 % in S-IMRT vs 17 % in Do-IMRT).

##### Statistical analysis

The primary endpoint will be compared between the two groups using a two-sample *t*-test or non-parametric Mann–Whitney test, depending on distribution of the composite scores. The primary analysis will be by intention-to-treat, including all patients with 12 month MDADI data. A p-value of <0.05 will be considered statistically significant. Analysis of covariance (ANCOVA) will be used to investigate other patient and clinical factors that could be associated with change in MDADI composite score from baseline to 12 months post- treatment. Chi-squared or Fisher’s exact test will be used to compare patients in both groups with deterioration of 10 points or more in the MDADI composite score.

##### Interim analysis

The anticipated trial recruitment duration is 2 years and it is therefore unlikely that sufficient data on the primary endpoint, either for efficacy or futility, will be available to close the trial early, on that basis alone. A close monitoring approach will be adopted to identify loco-regional recurrences (LRR), which will be reported in an expedited fashion by the treating centre. The number of LRR from the total number of patients who commenced trial treatment at that point will be tabulated by treatment group along with a p-value from Fisher’s exact test. The Independent Data Monitoring Committee (IDMC) will use this as guidance together with other emerging trial data to advise on any early cessation of the trial.

#### Quality assurance (QA)

Centres taking part in the trial will be required to successfully to complete the comprehensive Radiotherapy Trials Quality Assurance Group (RTTQA) IMRT credentialing programme in order to be approved to enter patients. This consists of pre-trial contouring and planning benchmark cases exercises together with prospective case reviews for at least the first 2 recruited patients in each centre [41]. A streamlining process for contouring, aiming to minimise QA repetition, exists for centres that have participated in other IMRT Head and Neck trials.

#### Trial organisation

The DARS trial was developed through a multidisciplinary collaboration between the ICR-CTSU and the Head and Neck Units of Royal Marsden Hospital NHS Foundation Trust (RMH), University Hospital Bristol, Norfolk and Norwich University Hospital NHS Trust; Division of Radiotherapy and Imaging of the Institute of Cancer Research (ICR); Speech and Language Therapy (SLT) Departments of RMH, City Hospitals Sunderland NHS Foundation Trust; Department of Physics of RMH and RTTQA. ICR-CTSU will have overall responsibility for trial co-ordination, data collation, central statistical monitoring of data and all interim analysis. A Trial Management Group will be responsible for the day to day running of the trial. The trial will be overseen by an independent Trial Steering Committee. An IDMC will regularly review emerging safety and efficacy data in confidence. The trial is sponsored by RMH and conducted in accordance with the Principles of Good Clinical Practice. This study is included on the National Institute for Health Research portfolio (NIHR number 19934).

## Discussion

The DARS trial is, to the best of our knowledge, the first RCT aiming to demonstrate that reducing the radiation dose to critical swallowing structures can safely improve long- term swallowing function and quality of life. The DARS trial design represents the efforts of a successful collaboration between UK H&N oncologists, physicists, clinical trialists, and SLTs, particularly within the context of a H&N primary CRT trial. Additionally, the association between clinicians and SLTs, both in DARS and the currently recruiting PATHOS trial, has huge potential for the future integration of routine swallowing outcome measures into UK clinical practice for patients with HNC. The trial methodology reflects the necessity to minimise confounding factors that might affect the robustness of its final results. Excluding patients with pre-existing dysphagia ensures that any post- treatment swallowing dysfunction is as a result of (chemo)-radiation treatment alone. Likewise, patients with posterior pharyngeal wall tumours are ineligible for the trial as any meaningful sparing of the PCM is unlikely to be achievable. By being selective in our approach, the trial is better equipped to determine the effectiveness of Do-IMRT across a homogeneous group of pharyngeal cancer patients. Defining the most accurate time point for long-term dysphagia remains controversial within the context of a clinical trial. Swallow sparing studies have so far adopted various time points ranging from 3 to 12 months following completion of treatment [4, 28, 36, 42]. While 3 months is clearly too early to assess long-term functional outcomes, patterns of swallowing function can change between 6 and 12 months with stabilisation thereafter [4, 43], suggesting that the 12- month timeline from treatment completion is more likely to be predictive of subsequent dysphagia.

The DARS trial recognises the importance of a comprehensive assessment for evaluating swallowing by including a multidimensional, longitudinal panel of functional outcome measures integrating instrumental, clinician- rated and patient- reported scales. The dashboard of swallowing measures adopted in DARS was developed in partnership with SLT leads from the PATHOS trial and should help the standardisation of future swallow outcome assessment and reporting. The MDADI composite score, the primary endpoint of DARS, is generated from a feasible and validated patient- reported swallow- specific questionnaire incorporating information from a patient’s physical, functional and emotional level at various recovery time points. Increasingly, it is being adopted as functional outcome tool for a number of head and neck cancer trials [44, 45].

**Fig. 2:**
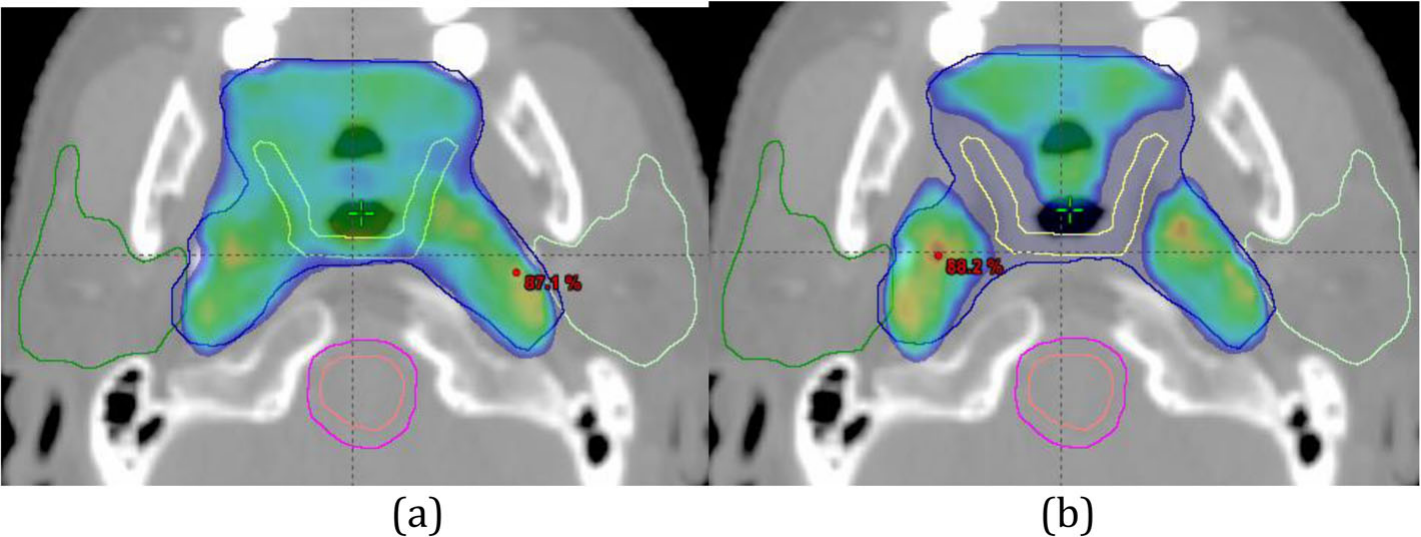
Tyler et al. [46] PTV_5400 (blue) dose distribution in (**a**) S- IMRT arm and (**b**) Do-IMRT, demonstrating sparing of dose to Plan SMPCM (yellow)

The Do-IMRT is an adaptation of the planning methodology used by Feng et al. in that it will strive to spare the part of the pharyngeal constrictors lying in the elective target volume CTV_5400 rather than the medial RPN alone (Fig. 2) [46]. A theoretical risk of increased recurrence in the spared tissue exists with this approach. It is noteworthy that similar concerns were raised with the landmark parotid- sparing IMRT trial (PARSPORT), but these were proven to be unfounded when the data were analysed [19]. Furthermore, it is well established that the majority of the local recurrences following primary radiation- based treatment are in the immediate vicinity of the primary tumour site (GTV + 1 cm). Importantly, there will be no compromise of target coverage in this volume with Do-IMRT [47–50]. Therefore, the risk of recurrence in the spared tissue is perceived to be minimal. Nevertheless, the IDMC will closely monitor loco-regional recurrence rates and advise on early stopping of the trial if this is deemed necessary. Mean doses to the constrictors above 50 Gy have previously been shown to be associated with the risk of long-term dysphagia [18, 51], and have consequently been incorporated as the dosimetric constraint for the PCM within the study.

In conclusion, reducing the risk of late dysphagia in pharyngeal cancers is vital to improve long-term HR-QoL. An increased understanding of the clinical and dosimetric relationship between the swallowing structures and radiotherapy- related dysphagia, together with the availability of novel IMRT techniques, makes it an optimal time to run the DARS trial. The trial is aiming to address the limitations of previous studies aiming to minimise dysphagia by testing Do-IMRT in a randomised study, crucially incorporating a multidimensional approach to swallowing assessment.

## Abbreviations

CRT: Chemoradiation
CRUK: Cancer Research UK
CTV: Clinical target volume
DARS: Dysphagia/aspiration at risk structures
DIGEST: Dynamic Imaging Grade of Swallowing Toxicity
Do-IMRT: Dysphagia-optimised intensity-modulated radiotherapy
FEES: Fibreoptic endoscopic evaluation of swallowing
GTV: Gross tumour volume
HPV: Human papillomavirus
HR-QoL: Health-related quality of life
ICR-CTSU: Institute of Cancer Research Clinical Trials Statistics Unit
IDMC: Independent Data Monitoring Committee
LRR: Locoregional recurrence
MDADI: MD Anderson Dysphagia Inventory
NIHR: National Institute for Health Research
NTCP: Normal tissue complication probability
OAR: Organs at risk
PATHOS: Post-operative adjuvant treatment for HPV positive tumours
PCM: Pharyngeal constrictor muscles
PSS-HN: Performance status scale-head and neck
PTV: Planning target volume
QA: Quality Assurance
RCT: Randomised controlled trial
RMH: Royal Marsden Hospital
RPN: Retropharyngeal lymph nodes
RTTQA: Radiotherapy Trials Quality Assurance
SGL: Glottic and supraglottic larynx
S-IMRT: Standard intensity-modulated radiotherapy
SLT: Speech and Language Therapist
UW-QoL: University of Washington Quality of Life Questionnaire
VF: Videofluoroscopy
WST: Water swallowing test
